# Digital replantation in forward surgical units: a cases study

**DOI:** 10.1051/sicotj/2018004

**Published:** 2018-03-16

**Authors:** Laurent Mathieu, Michel Levadoux, Emmanuel Soucany de Landevoisin, Tarun J. McBride Windsor, Sylvain Rigal

**Affiliations:** 1 Department of orthopedic traumatology reconstructive surgery, Percy Military Hospital, ﻿Clamart France; 2 Department of surgery, French Military Medical Academy, Ecole du Val-de-Grâce, Paris France; 3 Hand surgery unit, Saint-Roch private clinic, Toulon France; 4 Department of orthopedic surgery and traumatology, Laveran Military Hospital, Marseille France; 5 Department of thoracic surgery, Percy Military Hospital, Clamart France

**Keywords:** Austere environment, Digital amputation, Microsurgery, Military, Replantation

## Abstract

*Introduction*: Noncombat-related hand injuries are common in current theatres of operations. Crushing is one of the most frequent mechanisms that may cause traumatic amputations of digits. In the military setting, management of these digital amputations is challenging regarding limitation in microsurgical means in medical treatment facilities and aeromedical evacuation delays out of the combat zone.

*Methods*: Two cases of digital replantation performed in French forward surgical units are described. The first case was a complete distal amputation of the medius which was successfully replanted in the operating theatre of an aircraft carrier. No complication was observed after evacuation. Functional and aesthetic results were excellent. The second case was a ring finger avulsion revascularized in a role 2 facility in Central African Republic. Unfortunately, revascularization failed due to arterial thrombosis during evacuation.

*Results*: Digital, hand or more proximal upper extremity replantation may be considered for isolated amputations due to work-related accidents within the combat zone. For a surgeon trained to microsurgery, a microsurgical set and magnification loupes enable to attempt such procedures in austere conditions.

*Discussion*: The authors propose an algorithm of management in the field according to the type and level of amputation.

## Introduction

Hand injuries represent a large part of wartime upper extremity injuries and are associated with approximatively 10% of all aeromedical evacuations (MEDEVAC) from current theatres of operations [1–4]. Penn-Barwel et al. [3] established that casualties with isolated hand injuries represented 6.5% of all British casualties evacuated from Iraq and Afghanistan, with a clear majority of non-combat related injuries. Miller et al. [2] reported 4.4% of traumatic nonbattle hand injuries in the US troops treated at the lbn Sina hopsital, in Baghdad Iraq, with required MEDEVAC in 22% of the cases.

Crushing is the most frequent mechanism for these work-related injuries occurring because of closure of vehicle doors, hatches or turrets [1,2,4]. This mechanism can lead to partial or complete digital amputations which are fortunately uncommon. According to Brininger et al. [5] digital amputations represented less than 0.2% of the upper extremity injuries sustained by the US military during peacetime and wartime. Ideally, these work-related injuries should be treated in the same manner than in civilian practice following indications for digital replantation. Selection criteria for replantation procedures are now clearly defined for thumb, single digit, multiple digit and mid-palm amputations [6–9]. Despite limitations in microsurgical means within forward surgical units, it seems that replantation or revascularization could be performed for isolated hand injuries by orthopedic surgeons trained to microsurgery with magnification loupes. Usually digital replantation should not be attempted in cases of ballistic trauma, considering their severity and potential associated injuries or massive casualties situation [10].

The following cases describe the achievement of digital replantation for work-related crush injuries in forward surgical units with limited microsurgical capacity. Resulting from this experience, the authors propose an algorithm for management of noncombat-related digital or hand amputations in deployed medical treatment facilities (MTFs).

## Illustrative cases

### Case 1

**Figure 1:**
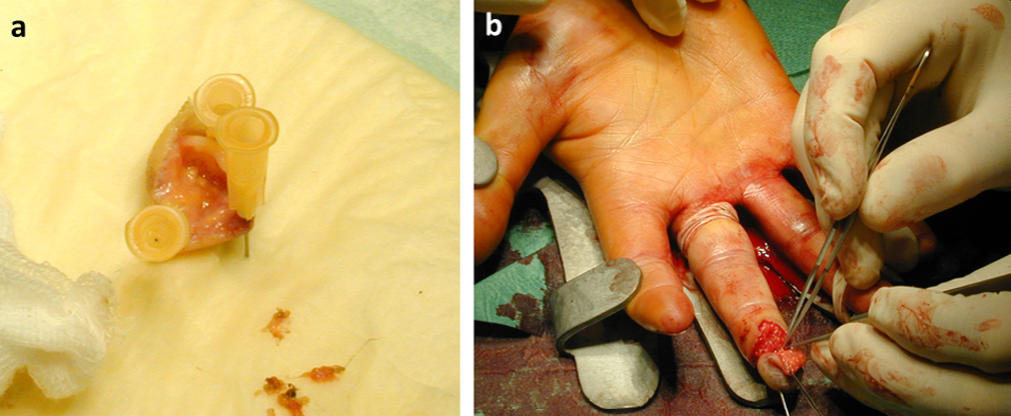
Preparation of amputated part (a) and replantation under magnification loupes (b).

**Figure 2:**
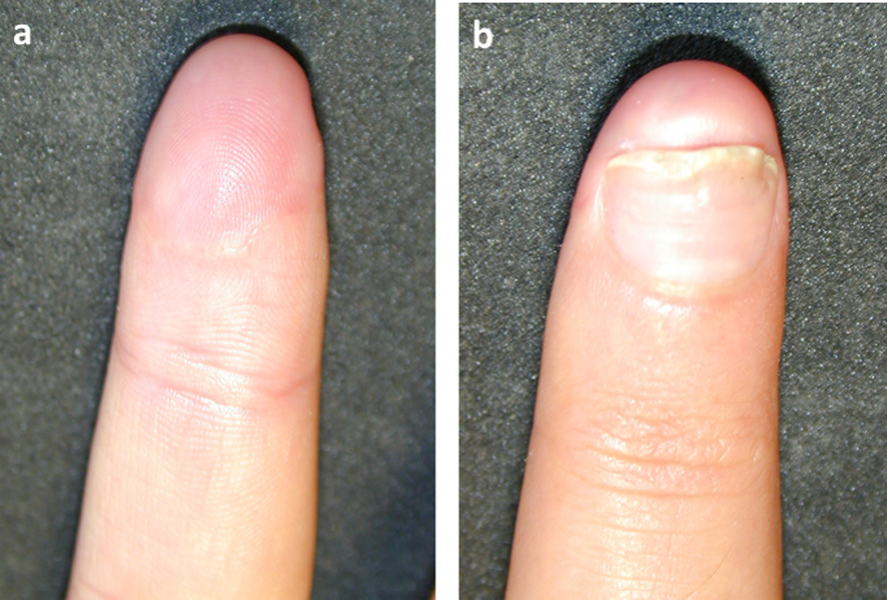
Volar (a) and dorsal (b) view of the replanted finger at the follow-up of 4 months.

A 25-year-old male serviceman sustained a crush injury of his right medius finger on the flight deck of the Charles De Gaulle aircraft carrier. He experienced oblique distal amputation through the nail bed in Ischikawa zone 1 [11]. An emergency replantation was decided by the onboard orthopedic surgeon who was also hand surgeon. In absence of operating microscope, the procedure was performed under ×3.5 magnification loupes with microsurgical instruments. While the anesthesiologist began locoregional anesthesia, the amputated fragment was prepared. Skin and subdermal tissue were gently debrided, the central artery was located and a 10/10 K-wire was placed using the «in-and-out» method to fix the distal phalanx. Next, the digital stump was debrided and trimmed. The proximal part of the central artery was dissected. After bony fixation, proximal and distal arterial targets were aligned and arterial repair was performed under tourniquet using interrupted stitches of 10-0 (Figure 1). No vein was suitable for anastomosis and nerve repair was unnecessary because the digital nerves had already trifurcated into distal branches. Several 5-0 nylon sutures were used to loosely approximate the skin. The nail bed was repaired with 6-0 Dexon sutures. For venous outflow, a stab incision was made at the pulp tip and the nail plate was removed. The wound was covered with a nonadherent dressing, the hand was not elevated and kept warm. To prevent vascular thrombosis 160 mg of acetylsalicylic was injected intravenously during surgery, and was continued per-os for 1 month [7]. Prophylactic antibiotic medication by penicillin with clavulanic acid was administrated for 3 days. The following day the patient was evacuated to a hand surgical unit in France where postoperative care was performed. A blood transfusion was required due to the postoperative external bleeding necessary to maintain venous outflow. After a follow-up period of 4 months, a new nail was growing with an excellent aesthetic aspect (Figure 2). Range of motion of the distal interphangeal joint was normal and bone union of the distal phalanx was acquired. Sensation recovery was incomplete with a static two-point discrimination of 7 mm.

### Case 2

**Figure 3:**
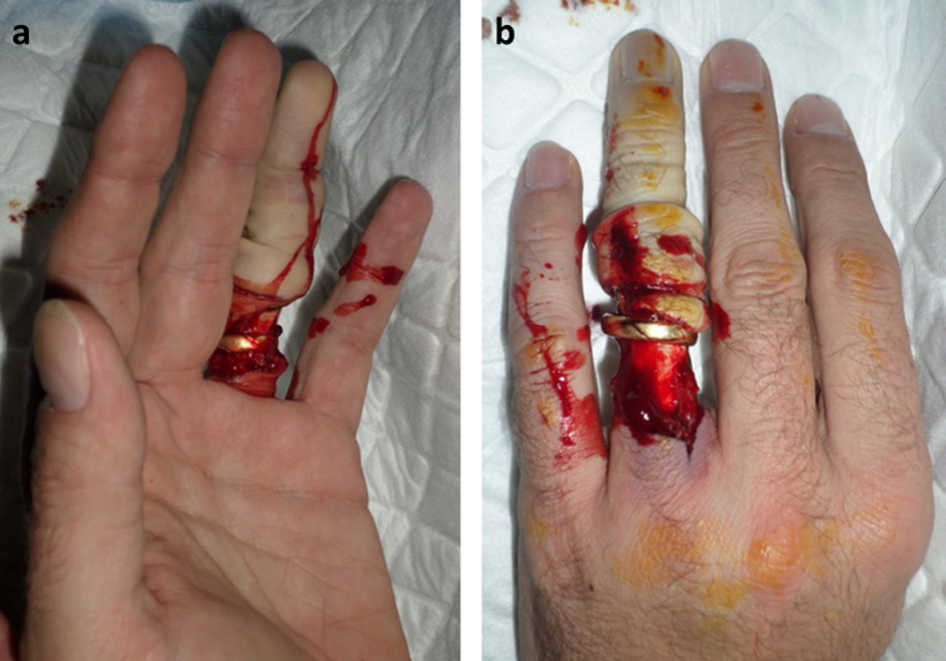
Volar (a) and dorsal (b) aspect of the ring avulsion injury.

**Figure 4:**
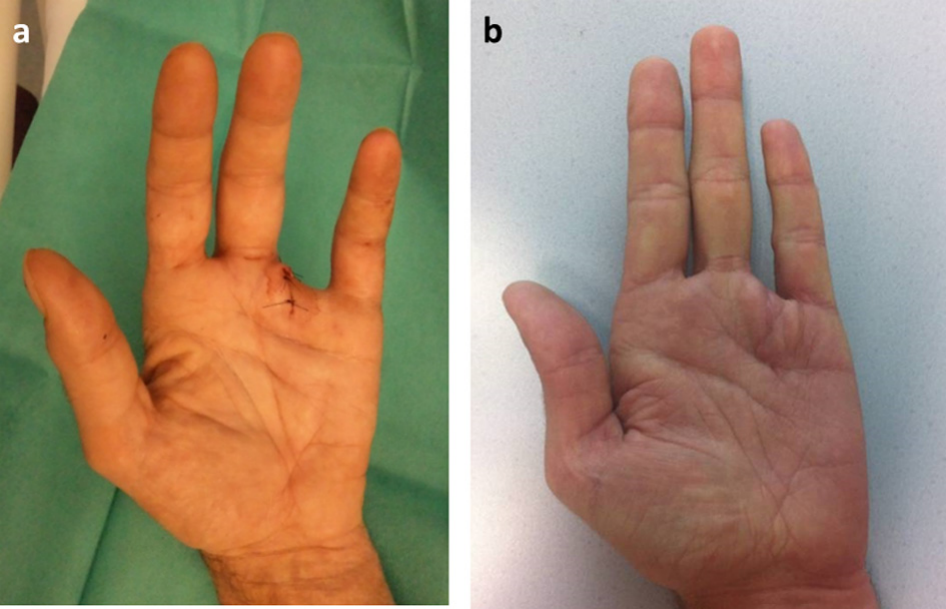
Temporary (a) and definitive (b) proximal amputation of the fourth ray.

A 42-year-old male soldier deployed in Central African Republic presented a ring avulsion injury of his left hand caused by a wedding band while he was unloading from a truck. The skin sleeve was disrupted at the proximal phalanx with ischemia due to complete avulsion of both neurovascular bundles, but the flexor tendons and bones were preserved (Urbaniak class I) [12] (Figure 3). Primary surgical management was performed in a forward surgical team (FST, role 2 MTF). Considering the situation of warm ischemia with impossibility for MEDEVAC within 6 h a temporary revascularization was attempted by the orthopedic surgeon who was not hand surgeon. The skin and subdermal tissue were first debrided. As the arteries were transected at various levels, a direct arterial anastomosis crossed from one side to the other was decided. After preparation of arterial ends the proximal ulnar digital artery was anastomosed to the distal radial digital artery using 9.0 nylon sutures with microsurgical instruments and ×2.5 magnification loupes. A venous bypass was not performed because the surgeon was not familiar with this procedure. Dorsal veins and nerves were not repaired. Loose skin closure and a stab incision made at the pulp tip permit venous outflow. At the end of the procedure the color of the finger, pulp capillary refill and skin temperature indicated good arterial perfusion. During surgery, the patient received 2 g of penicillin with clavulanic acid and 160 mg of acetylsalicylic intravenously. He was evacuated to France few hours later and arrived at the Percy military hospital (role 4 MTF) more than 24 h after the trauma. Unfortunately, an arterial thrombosis occurred during transportation and a distal digital necrosis was noticed on arrival. An early regularization at the metacarpophangeal joint level was required. One month later, a definitive proximal amputation was performed with quick return to duty (Figure 4). At the last follow-up of 12 months, function and cosmetic appearance were optimal. The patient was waiting for a next tour. He was satisfied and grateful for the efforts made to save his finger.

## Discussion

To the author's knowledge, no case of digital replantation performed in the combat zone has been reported before. Management of work-related digital amputations is highly challenging in this setting due to the lack of operative microscope and hand surgeon in most of forward MTFs. Even if small sized portable microscopes were available, only surgeons trained to microsurgery could achieve such procedures. This uncommon topic represents one the limitations of the medical support in current theatres of operations and may cause medico-legal matters related to the notion of «loss of chance».

Management of hand injuries is often challenging in the combat zone. Even simple flexor tendon or nerve transections can be difficult to treat for a non-specialized surgeon and may jeopardize hand function [4,13]. To achieve an optimal functional outcome, several authors recommend that definitive treatment of patients with tendon and nerve injuries (or with hand fracture requiring surgery) should be done in hand surgical units after MEDEVAC [1,3,14]. Initial management should be limited to meticulous *débridement* with precise wound assessment to avoid infection and to prepare the hand for secondary repair according to the hand damage control orthopedics (DCO) principles [13]. However, hand fractures and tendon repairs are frequently performed by orthopedic or plastic surgeons in forward MTFs, especially when evacuation must be delayed for operational constraints [4,15]. Application of hand DCO procedures is also limited in cases of vascular damage with ischemia: revascularization of digital amputations or ring avulsion injuries can hardly be delayed.

French orthopedic surgeons are trained to hand surgery and microsurgery during their initial instruction period, and many of them treat daily patients with hand trauma in military hospitals. Only patients with complex hand injuries, including mangled extremity or digital amputation requiring replantation, are referred to civilian specialized hand surgery units. When deployed these surgeons usually treat local patients with complex injuries using a microsurgical instrument set and magnification loupes. Despite a substantial risk of failure due to the absence of operative microscope, most of them can attempt a revascularization at the hand level when the situation does not allow for evacuation to specialized units in appropriate time.

This cases study shows that digital replantation can be performed, or at least attempted, in the combat zone following almost the same principles than in civilian practice. General indications for replantation include thumb amputations, multiple digits amputations, single digit amputations distal to the insertion of the flexor digitorum superficialis, and children whatever the level of amputation [6,7]. Ring avulsion injuries are also good indications as long as the flexor tendons remain intact, because if the replantation procedure is successful, the digit will become immediately functional [6]. In contrast, replantation is contraindicated for single digit amputation at the level of the proximal phalanx or at the proximal interphalangeal joint, for severely crushed or avulsed digits with extensive soft tissue defect, for segmental injuries at multiple levels, and in patients with associated life-threatening injuries [6]. These recommendations explain why replantation is not indicated for hand combat-related injuries caused by projectile or explosive devices, and then, why microsurgical means are often missing in forward MTFs.

**Figure 5:**
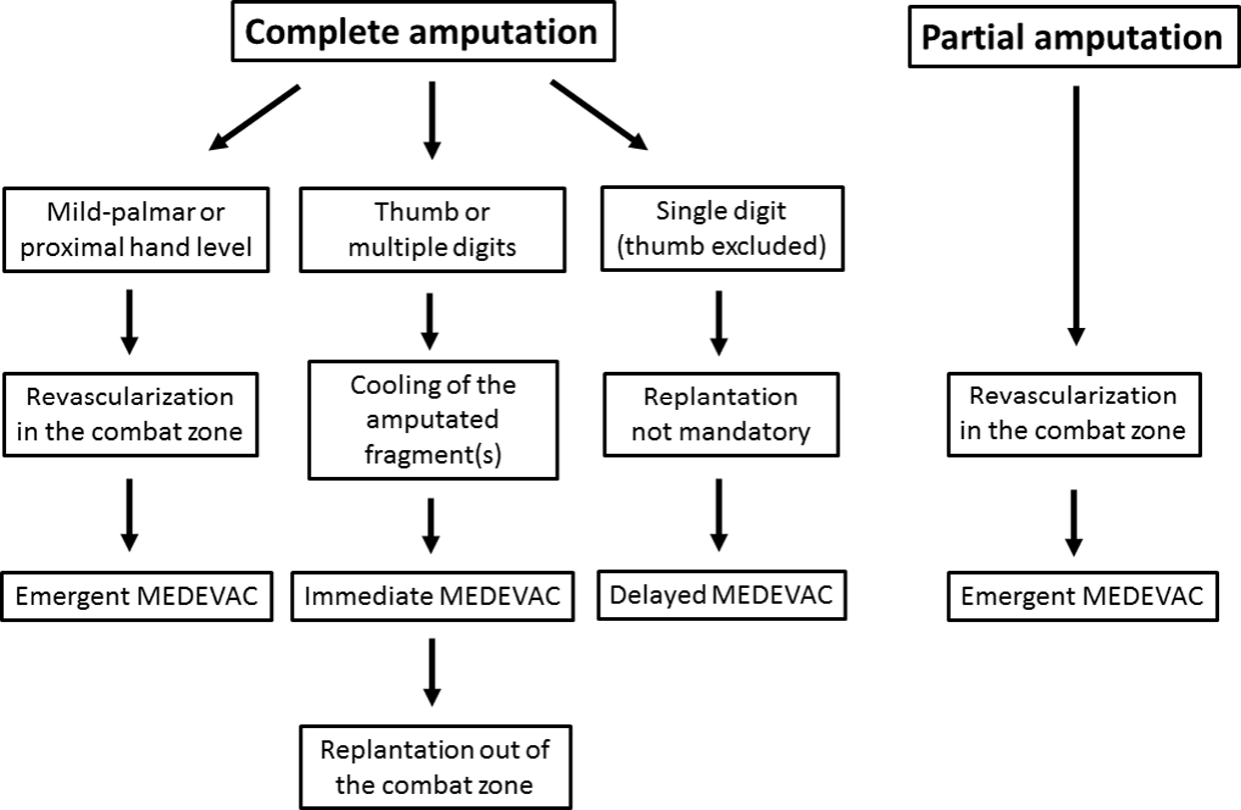
Proposed algorithm for management of digital or hand amputations in forward MTFs.

Ischemia is a key factor in determining the success of replantation. Muscle begins to undergo irreversible changes after 6 h at room temperature, but since digits contain no muscles, warm ischemia is tolerated for a longer period [6]. According to Soucacos et al. [6] the time allowed for warm ischemia at the digit level is about 8 h, compared to 6 h for the upper or lower extremity. They also found that by cooling the amputated segment to 4 °C the ischemia time for digits can be extended to up to 30 h [6]. Thus, MEDEVAC of patients with a complete digital amputation is feasible if the amputated part is carefully cooled during transportation. To the opposite, patients with partial digital amputations or ring avulsion injuries cannot be evacuated before revascularization has been performed. The aim is not to achieve a complete and definitive treatment (nerve repair is not expected) but only to restore sufficient arterial perfusion to permit evacuation out of the combat zone. Based on this experience, we proposed an algorithm for management of non-combat related digital amputations in the combat zone (Figure 5).

Patients with complete thumb or multiple digits amputations should benefit from ideal replantation after preservation of the amputated part(s) at 4 °C and immediate MEDEVAC to the nearest hand surgical unit. Replantation should be performed as soon as possible but can be delayed of 24 to 30 h. Proper storage of amputated fragment is crucial to ensure replantation success, especially during an extended aeromedical evacuation. First, it should be rinsed in normal saline or lactated Ringer's solution to remove gross contamination, and then wrapped in moist gauze. The wrapped fragment should be placed in a waterproof bag, which is placed in a container of ice and water. It is important that the fragment not be placed directly on ice, or placed on dry ice, because these methods risk freezing the tissue causing irreversible tissue damage [8,16].

Replantation of a single digit distal amputation is more questionable in this setting, as the loss of a single digit (except the thumb) has a limited effect on hand function [17,18]. Furthermore, distal replantation is technically challenging. In case n°1 the decision was made not to evacuate the patient. Replantation was performed on the aircraft carrier because an experienced hand surgeon was present. Nail salvage permitted an optimal functional and cosmetic outcome, but amputation closure using a local flap would have been a valid alternative [19]. However, this case demonstrates that digital arterial anastomoses are feasible in austere conditions using magnification loupes and basic microsurgical instruments.

Patients with partial digital amputations, such as ring avulsion injuries, or patients with complete mid-palm or more proximal amputations cannot benefit from delayed replantation in specialized units. Warm and muscle ischemia compels to perform revascularization in the combat zone within 6–8 h. Patients should be informed that the risk of failure is high but that there is no better option. Revascularization of avulsion or crushing injuries is particularly challenging because intimal lesions may cause thrombosis in the following hours as observed in case n°2. Ring avulsion injuries usually require venous bypass which are almost impossible to perform in the field for non-specialized surgeons. Furthermore, the risk of thrombosis of arterial anastomosis may be increased by vasospasm and blood pressure changes occurring during air transportation.

## Conclusion

Battlefield medical support is not adapted to management of work-related digital or hand amputations. Cooling of amputated fragment(s) and immediate MEDEVAC to a specialized hand surgery unit out of the combat zone should be considered for thumb or multiple digits amputations. In cases of proximal hand amputations or digital warm ischemia, revascularization should be attempted on the field despite limited microsurgical means.

## Conflict of interest

The authors declare no conflict of interest in relation with this paper.

## Disclaimer

The views expressed in this manuscript are those of the authors and do not reflect the official policy or position of the French Medical Health Service.
